# Self-Reported Prevalence of Chronic Diseases and Their Relation to Selected Sociodemographic Variables: A Study in INDEPTH Asian Sites, 2005

**Published:** 2008-06-15

**Authors:** Hoang Van Minh, Nawi Ng, Sanjay Juvekar, Abdur Razzaque, Ali Ashraf, Abdullahel Hadi, Kusol Soonthornthada, Uraiwan Kanungsukkasem, Tran Huu Bich, Peter Byass

**Affiliations:** FilaBavi Demographic Surveillance Site; Purworejo Demographic Surveillance Site, Indonesia; Vadu Demographic Surveillance Site, India; Matlab Demographic Surveillance Site, Bangladesh; HSID Demographic Surveillance Site, Bangladesh; WATCH Demographic Surveillance Site, Bangladesh; Kanchanaburi Demographic Surveillance Site, Thailand; Kanchanaburi Demographic Surveillance Site, Thailand; Chililab Demographic Surveillance Site, Vietnam; Umeå International School of Public Health, Umeå University, Umeå, Sweden

## Abstract

**Introduction:**

Lack of reliable population-based data, especially morbidity data, is a barrier to preventing and controlling chronic diseases in developing countries. We report the self-reported prevalences of major chronic diseases in Southeast Asia and examine their relation to selected sociodemographic variables in adults.

**Methods:**

Data are from a 2005 cross-site study of 8 sites in 5 Asian countries that surveyed 18,484 people aged 25–64 years. Respondents were asked whether they had been told by a health care worker that they had any of 7 chronic health conditions: joint problems, stroke, heart disease, diabetes, pulmonary disease, hypertension, or cancer. Information about participants' sex, age, and educational level was also obtained.

**Results:**

We found that 22.7% of men and 31.6% of women reported having at least 1 of the chronic health conditions of interest, and 5.1% of men and 9.2% of women reported having 2 or more chronic conditions. Multivariate regression analyses showed that women had more chronic conditions than men, the prevalence of chronic conditions increased with age, and people with the least education were more likely to have chronic conditions.

**Conclusion:**

Chronic conditions are commonly reported among adults in Asian countries. Disparities in the prevalence of chronic conditions by sex and education are evident.

## Introduction

Chronic diseases are leading causes of morbidity and mortality worldwide (1-5). In 2005, of 58 million all-cause deaths worldwide, 35 million were attributable to chronic diseases, and 80% of those were in developing countries (3). Chronic diseases kill more middle-aged people in poorer than in wealthier countries, and chronic diseases affect 5 times as many people as human immunodeficiency virus/AIDS does in developing nations (3). Because health care systems in developing countries usually are designed to manage acute communicable diseases, the increasing prevalence of chronic disease is a major challenge (3,5-9).

Mortality from chronic diseases is also increasing in Asia (10,11); however, lack of reliable population-based data, especially morbidity data, is a barrier to preventing and controlling chronic diseases in these countries. Thus far, little attention has been paid to the problem, particularly with regard to conducting surveillance to monitor trends over time and across populations (3,12).

To provide information about epidemiologic aspects of chronic diseases in Asian countries, we report the self-reported prevalence of major chronic conditions and examine their relation to selected sociodemographic factors among adults from 8 demographic surveillance system (DSS) sites in 5 Asian countries.

## Methods

### Data source

INDEPTH (International Network for Demographic Evaluation of Populations and Their Health) (www.indepth-network.org) is an international network of DSS sites founded to link existing demographic field sites. The objectives of INDEPTH are to 1) initiate cross-site, longitudinal health and social studies in severely resource-constrained populations; 2) disseminate findings among external stakeholders to maximize impact on policy and practice; 3) foster and support capacity building and cross-site collaborations among INDEPTH member sites; and 4) facilitate the process for donors to fund health and social research projects in the developing world, especially in Africa and Asia (13). Each DSS site continually measures demographic and health variables among the population of a geographically defined area (13,14).

In 2005, we conducted a cross-site study in 8 DSS sites in 5 Asian countries: FilaBavi and Chililab in Vietnam; Health System and Infectious Disease (HSID), Woman Abuse Tracking in Clinics and Hospitals (WATCH), and Matlab in Bangladesh; Kanchanaburi in Thailand; Purworejo in Indonesia; and Vadu in India. A representative sample of approximately 1000 men and 1000 women aged 25 to 64 years was randomly selected from existing DSS sampling frames and surveyed at each site, except at HSID, where the sample was approximately 2000 men and 2000 women. The total surveyed was 18,484. Data were collected through personal household interviews conducted by trained field workers. A standardized structured questionnaire was used at all sites to collect information about chronic conditions and sociodemographic variables. Field supervisors and the investigators of this study controlled data quality in the field.

### Self-reported chronic conditions

Respondents were asked if they had ever been told by a health care worker that they had any of 7 chronic health conditions: joint problems, stroke, heart disease, diabetes, pulmonary disease, hypertension, or cancer. Other conditions, such as kidney disease, skin disease, or psychological disorders, were not included because the prevalence of those diseases in the study population is low. We calculated the total number of chronic conditions by adding the number of positive responses to these questions.

### Sociodemographic variables

Sex, age, and educational level were recorded for each participant. Education was used as a proxy for socioeconomic status, which has several advantages in our settings. Education tends to determine occupation and is usually stable after young adulthood. Unlike occupational class, education allows classification of persons who do not work. Educational status is an individual measure of socioeconomic position and may be a better indicator than those based on household measures, such as household income, which are difficult to measure at our sites. In addition, classification of educational status, based on the highest level of schooling, was comparable between countries. We categorized educational level into 4 groups: fewer than 6 years of education, graduate of primary school (completion of grade 6), graduate of secondary school (completion of grade 9), and tertiary education (completion of grade 12) and higher.

### Statistical methods

We calculated descriptive and analytical statistics with Stata 8 (StataCorp LP, College Station, Texas). Prevalences of different chronic conditions were calculated for each site and for all sites combined. Multivariate logistic regression and linear regression modeling were performed to examine the probability of developing chronic conditions in relation to sociodemographic variables at all sites. To account for different sampling methods between sites, we introduced sampling weights on calculations and regression analyses. Results were considered significant at *P* < .05.

### Ethical considerations

The study protocol was approved by the scientific board of the INDEPTH network and was in accordance with the ethics codes of each site. Informed consent was obtained from all participants before data were collected, and participants had the right to withdraw from the study at any time.

## Results

We surveyed 9241 men and 9253 women (Table 1). At all sites and for both sexes, the 55- to 64-year age group had the smallest proportion of respondents. Educational level varied greatly between sites; respondents in Bangladesh had the least education, and those in Vietnam had the most. Women were less educated at all sites, especially at the Bangladeshi sites and at Vadu in India.

Chronic conditions were commonly reported among respondents, especially among women (Table 2). Joint problems and hypertension were most commonly reported, whereas stroke and cancer were least common. The prevalence of chronic conditions varied considerably across sites: the proportion of people with at least 1 chronic condition was highest in Matlab (Bangladesh) and lowest in Purworejo (Indonesia). Approximately 5% of men and 9% of women reported having 2 or more chronic conditions (Table 3). The proportion of people with 2 or more self-reported chronic conditions was also highest in Matlab and lowest in Purworejo.

Multivariate logistic regression analyses of the effects of sociodemographic variables on chronic conditions showed that the prevalence of self-reported chronic conditions increased with age and differed significantly for all reported conditions (Table 4). However, the difference in prevalence of cancer was significant only among people aged 55 to 64 compared with those aged 25 to 34. People with the lowest educational levels were more likely to report any chronic condition than were those with higher education.

The effects of sociodemographic variables on total of number of chronic conditions were examined by multivariate linear regression (Table 5). Women, older people, and respondents with less education were more likely to have more chronic conditions, although the difference in the number of conditions by education was significant only for those who graduated from primary school compared with those with fewer than 6 years of schooling.

## Discussion

This study provides the first overview of the magnitude of self-reported chronic conditions at 8 INDEPTH Asian sites. The overall self-reported prevalence of these conditions (22.7% in men and 31.6% in women) indicates that they already affect a large proportion of the population in the study sites. These figures are lower than the overall prevalence of 38% found in China (15), 43% in Japan (16), and 77% in the United States (17).

Women at our sites reported more chronic conditions than did men. Although men and women may perceive, evaluate, and act on symptoms differently (18), many studies have shown no differences by sex in the reporting of chronic conditions (19,20). Our study probably indicates health disparities by sex in Asian countries, which suggests the need for more attention to women's health and health care in these countries. We also found that increasing age is associated with higher self-reported prevalence of chronic conditions, which is in concordance with other findings (8,21-23). The self-reported prevalence of chronic conditions in our study was significantly higher among respondents with less education, even after adjusting for age and sex; this finding is similar to those of other studies that showed inverse socioeconomic gradients in chronic diseases (16,22-24).

Some methodologic limitations of this study may affect interpretation of the results. The self-reported prevalence of chronic conditions is subject to recall bias and may not reflect the true prevalence; reporting also can be affected by respondents' knowledge, manifestations of the illness in everyday life, their willingness to report the condition, and frequency of contact with a physician (23). However, self-reports of several chronic conditions are substantially accurate when compared with physician diagnoses (25-27). Other factors that may contribute to the differences we observed are response rates, differences in age, and variability in definitions of chronic conditions. An additional limitation is that this cross-site study was not designed primarily to assess social patterns of chronic conditions at each respective site; therefore, comparing disease inequalities between sites and countries is problematic. This study, however, provides a broad overview of general patterns throughout all sites.

Given our findings, actions to reduce levels of chronic diseases in the studied settings are needed. Interventions should focus on disadvantaged groups, especially women and people with lower education. Knowledge of the prevalence and social patterning of chronic diseases is likely to be useful for health professionals in predicting health trends and directing health planning and interventions. Further study, as part of the ongoing INDEPTH surveillance, will help provide high-quality and timely data to increase the effectiveness of interventions and reduce the burden of chronic disease in Southeast Asia.

## Figures and Tables

**Table 1:** Age and Educational Level of Participants From 8 Demographic Surveillance System Sites in 5 Asian Countries, 2005^a^

Characteristic	All Sites	Vietnam		Bangladesh			Indonesia	India	Thailand
		C	F	H	W	M	P	V	K
**Men**									
**No.**	9241	1096	993	2016	1000	1047	993	1039	1057
**Age, y, %**									
25–34	30.7	29.9	31.4	34.0	37.5	30.1	22.5	32.0	28.7
35–44	30.7	31.2	32.5	27.1	29.1	32.3	29.5	33.0	31.2
45–54	24.7	27.2	24.4	23.9	21.5	23.1	28.6	21.6	25.4
55–64	14.0	11.8	11.8	15.0	12.0	14.5	19.4	13.4	14.7
**Education, %**									
<6 years	31.4	5.4	8.0	48.2	60.9	48.4	22.3	25.7	28.6
Primary school	22.4	14.1	17.3	9.4	12.4	26.7	36.8	11.8	44.6
Secondary school	24.5	41.5	57.9	30.6	11.9	6.0	18.7	20.2	11.1
Tertiary school or university	21.7	38.9	16.8	11.8	14.8	19.0	22.2	42.2	15.7
**Women**									
**No.**	9253	1108	1023	2007	1000	1026	958	1041	1090
**Age, y, %**									
25–34	32.0	29.1	30.4	34.8	44.6	34.5	20.2	31.9	28.6
35–44	30.7	30.5	31.6	29.8	26.8	32.1	32.4	31.2	31.6
45–54	22.9	27.8	25.2	21.8	17.0	18.5	26.3	22.4	25.2
55–64	14.4	12.6	12.8	13.6	11.6	14.8	21.1	14.5	14.5
**Education, %**									
<6 years	42.2	10.4	11.7	65.5	75.3	59.7	30.8	56.3	39.1
Primary school	25.3	19.8	19.9	11.2	14.6	28.8	39.5	10.7	45.4
Secondary school	20.7	41.4	54.3	20.0	6.6	5.4	16.4	17.0	6.0
Tertiary school or university	11.8	28.4	14.1	3.3	3.5	6.1	13.2	16.0	9.5

a The 8 demographic surveillance system sites are Chililab (C), FilaBavi (F), Health System and Infectious Disease (H), Woman Abuse Tracking in Clinics and Hospitals (W), Matlab (M), Purworejo (P), Vadu (V), and Kanchanaburi (K).

**Table 2:** Prevalence of Selected Chronic Conditions Among 8 Demographic Surveillance System Sites in 5 Asian Countries, 2005^a^

Chronic Condition	Site, %								

	All Sites	Vietnam		Bangladesh			Indonesia	India	Thailand
		C	F	H	W	M	P	V	K
**Men (n = 9241)**									
Any chronic condition	22.7	21.5	29.8	31.8	13.5	34.4	10.7	28.6	21.2
Joint problem	10.7	6.2	9.8	16.3	92.5	20.7	0.8	19.3	4.1
Stroke	1.3	0.6	1.2	2.0	0.5	1.6	0.6	1.7	1.4
Heart disease	5.0	6.5	5.3	6.4	66.3	5.3	0.6	1.1	3.3
Diabetes	2.2	0.7	0.5	3.5	35.4	2.8	1.4	1.7	3.0
Pulmonary disease	6.5	6.5	14.7	3.3	82.4	6.5	1.2	2.1	4.1
Hypertension	7.4	7.1	5.6	10.4	9.9	6.7	6.9	3.2	9.4
Cancer	0.5	0.2	0.1	0.9	50.0	1.0	0.2	0.2	0.5
**Women (n = 9253)**									
Any chronic condition	31.6	24.8	35.5	45.8	24.0	49.2	15.6	35.8	30.8
Joint problem	17.4	9.7	15.4	28.3	8.9	30.6	1.6	28.8	9.2
Stroke	1.1	0.6	0.7	1.5	0.7	1.8	0.3	2.2	0.6
Heart disease	7.6	8.8	6.7	7.8	77.7	10.7	1.9	1.1	6.1
Diabetes	2.3	0.4	0.2	3.1	40.9	4.2	1.0	1.0	3.6
Pulmonary disease	6.1	6.0	15.4	3.4	94.8	5.4	1.4	2.5	2.3
Hypertension	11.6	5.5	4.1	18.9	24.2	14.6	10.6	4.5	15.0
Cancer	2.1	0.8	1.0	3.5	100.0	3.0	0.6	0.2	2.4

a The 8 demographic surveillance system sites are Chililab (C), FilaBavi (F), Health System and Infectious Disease (H), Woman Abuse Tracking in Clinics and Hospitals (W), Matlab (M), Purworejo (P), Vadu (V), and Kanchanaburi (K).

**Table 3:** Number of Self-Reported Chronic Conditions in Participants From 8 Demographic Surveillance System Sites in 5 Asian Countries, 2005^a^

No. Chronic Conditions	Site (%)								

	All Sites	Vietnam		Bangladesh			Indonesia	India	Thailand

		C	F	H	W	M	P	V	K
**Men (n = 9241)**									
0	77.3	78.5	70.2	68.2	86.5	65.6	89.3	71.2	78.8
1	17.7	16.2	23.8	23.1	11.0	26.1	9.4	24.4	15.9
2	3.9	4.1	4.7	6.1	1.5	6.4	1.3	3.7	4.7
3	1.0	1.1	1.2	2.1	0.8	1.6	0.0	0.5	0.5
≥4	0.2	0.1	0.1	0.6	0.2	0.4	0.0	0.1	0.1
**Women (n = 9253)**									
0	68.4	75.2	64.6	54.2	76.0	50.8	84.4	64.1	69.2
1	22.4	18.4	28.5	29.4	16.3	31.7	13.9	28.2	20.9
2	7.0	5.3	5.8	12.2	5.2	13.1	1.5	6.7	7.4
3	1.6	0.9	1.1	2.9	1.5	3.4	0.1	0.6	1.8
≥4	0.6	0.2	0.1	1.2	1.0	0.9	0.1	0.3	0.6

a The 8 demographic surveillance system sites are Chililab (C), FilaBavi (F), Health System and Infectious Disease (H), Woman Abuse Tracking in Clinics and Hospitals (W), Matlab (M), Purworejo (P), Vadu (V), and Kanchanaburi (K).

**Table 4:** Multivariate Logistic Regression Analyses of Effects of Sociodemographic Variables on Chronic Conditions Among Participants From 8 Demographic Surveillance System Sites in 5 Asian Countries, 2005^a^

Condition	Odds Ratio (95% Confidence Interval)^b^						

	Men	Age (y)			Education		

		35–44	45–54	55–64	Primary School	Secondary School	Tertiary School or University
Any chronic condition	0.61 (0.57–0.66)^c^	1.57 (1.40–1.76)^c^	2.49 (2.23–2.78)^c^	3.64 (3.25–4.07)^c^	0.89 (0.81–0.99)^c^	1.04 (0.94–1.15)	1.01 (0.9–1.13)
Joint problem	0.62 (0.56–0.69)^c^	1.43 (1.21–1.69)^c^	2.05 (1.75–2.41)^c^	2.73 (2.33–3.19)^c^	0.5 (0.43–0.57)^c^	0.48 (0.42–0.55)^c^	0.46 (0.38–0.54)^c^
Stroke	1.22 (0.91–1.64)	2.14 (1.17–3.91)^c^	3.52 (2.01–6.18)^c^	5.74 (3.31–9.96)^c^	0.76 (0.51–1.13)	0.93 (0.64–1.37)	0.57 (0.35–1.29)
Heart disease	0.62 (0.54–0.72)^c^	1.29 (1.02–1.64)^c^	1.71 (1.36–2.15)^c^	2.09 (1.66–2.62)^c^	0.98 (0.80–1.20)	1.15 (0.95–1.39)	1.11 (0.88–1.40)
Diabetes	0.95 (0.76–1.18)	2.10 (1.25–3.53)^c^	4.46 (2.73–7.27)^c^	6.77 (4.18–10.90)^c^	1.02 (0.77–1.36)	0.66 (0.48–0.92)^c^	1.13 (0.81–1.57)
Pulmonary disease	1.03 (0.89–1.20)	1.21 (1.11–1.44)^c^	1.49 (1.20–1.86)^c^	2.05 (1.66–2.55)^c^	0.79 (0.64–0.97)^c^	1.56 (1.30–1.88)	0.99 (0.78–1.26)
Hypertension	0.61 (0.55–0.68)^c^	1.69 (1.37–2.08)^c^	3.03 (2.49–3.68)^c^	4.32 (3.56–5.25)^c^	0.92 (0.79–1.06)	0.77 (0.66–0.90)^c^	0.95 (0.79–1.13)
Cancer	0.26 (0.18–0.38)^c^	1.47 (0.85–2.51)	1.32 (0.77–2.26)	1.67 (1.18–2.38)^c^	0.44 (0.29–0.68)^c^	0.32 (0.19–0.52)^c^	0.52 (0.3–0.91)^c^

a The 8 demographic surveillance system sites are Chililab and FilaBavi in Vietnam; Health System and Infectious Disease, Woman Abuse Tracking in Clinics and Hospitals, and Matlab in Bangladesh; Purworejo in Indonesia; Vadu in India; and Kanchanaburi in Thailand.

b Reference group for men is women; for age, 25–34 years; and for education, <6 years.

c Statistically significant at *P* < .05.

**Table 5:** Multivariate Linear Regression Analyses of Effects of Sociodemographic Variables on Total Number of Chronic Conditions in Participants from 8 Demographic Surveillance System Sites in 5 Asian Countries, 2005^a^

**Characteristic^b^**	Coefficient	95% Confidence Interval
**Sex**		
Male	−0.15^c^	−0.17 to −0.13
**Age, y**		
35–44	0.12^c^	0.09 to 0.14
45–54	0.26^c^	0.23 to 0.29
55–64	0.41^c^	0.38 to 0.44
**Education**		
Graduated from primary school	−0.03^c^	−0.05 to −0.01
Graduated from secondary school	0.01	−0.03 to 0.02
Graduated from tertiary school or university	0.01	−0.04 to 0.03
**Constant**	0.29	0.26 to 0.31

a The 8 demographic surveillance system sites are Chililab and FilaBavi in Vietnam; Health System and Infectious Disease, Woman Abuse Tracking in Clinics and Hospitals, and Matlab in Bangladesh; Purworejo in Indonesia; Vadu in India; and Kanchanaburi in Thailand.

b Reference group for sex is female; for age, 25–34 years; and for education, <6 years.

c Statistically significant at *P* < .05.
